# Supplementary material for: Cryo-electron tomography reveals the microtubule-bound form of inactive LRRK2
Source: eLife. 2025 Oct 31;13:RP100799. doi: 10.7554/eLife.100799 (PMC12578439; doi:10.7554/eLife.100799)
Supplement: Supplementary file 1. [file elife-100799-supp1.docx]

# Supplementary File 1: Cryo-ET data collection and model statistics

|  | #1 LRRK2^I2020T^+MLi-2, stepwise polymerization  (EMDB-45591: LRRK2)  (EMDB-45592: microtubule)  (EMDB-45593: WD40:ARM/ANK interface)  (PDB 9CHO) | #2 LRRK2^I2020T^+MLi-2, co-polymerization  (EMDB-45594: LRRK2) | #3 LRRK2 + MLi-2  (EMDB-45595: LRRK2) | #4 LRRK2 + GZD-824  (EMDB-45596: LRRK2) |
| --- | --- | --- | --- | --- |
|  | **Data collection and processing** | | | |
| Magnification | 42k | | | |
| Voltage (kV) | 300 | | | |
| Total dose (e–/Å^2^) | 120 | | | |
| Defocus range (μm) | -3.0 to -5.0 | | | |
| Defocus increment | 0.5 | | | |
| Acquisition scheme | Dose-Symmetric, -54/54, 3° step, group 3 | | | |
| Pixel size (Å) | 2.161 | | | |
| No. of frames | 8 | | | |
| # of tomograms | 131 | 150 | 196 | 143 |
| # of subtomograms | 65,612 | 83,007 | 45,052 | 70,363 |
| Final particle # | 60,556 | 45,155 | 42,687 | 53,214 |
| Symmetry imposed | C2 for LRRK2 WD40 dimer, C1 for symmetry expanded LRRK2,  -27.7° rot and 9.4 Å rise for microtubule | | | |
| Map resolution (Å)  FSC threshold = 0.143 (Å) | 7.8 (LRRK2),  5.9 (microtubule),  8.6(WD40:ARM/ANK) | 8.3 (LRRK2), | 8.3 (LRRK2) | 8.1 (LRRK2) |
| Map resolution range (Å) | 7 to >9 | 7.5 to >9.5 | 7.5 to >9.5 | 7 to >9 |
|  | **Refinement** | | | |
| Initial model used (PDB code) | 7LHT, 8TZH |  |  |  |
| Model resolution (Å)  FSC threshold = 0.143 (Å) | 7.8 (LRRK2) |  |  |  |
| Model resolution range (Å) | n/a |  |  |  |
| Map sharpening *B* factor (Å^2^) | -653 (LRRK2) |  |  |  |
| Model composition  Non-hydrogen atoms  Protein residues  Ligands | 26,693  1,792 MLi-2:1, GDP:1 |  |  |  |
| *B* factors (Å^2^)  Protein  Ligand | 471.87  N/A |  |  |  |
| R.m.s. deviations  Bond lengths (Å)  Bond angles (°) | 0.021  1.419 |  |  |  |
| Validation  MolProbity score  Clashscore  Poor rotamers (%) | 2.20  14.23  0.06 |  |  |  |
| Ramachandran plot  Favored (%)  Allowed (%)  Disallowed (%) | 90.17  9.83  0.00 |  |  |  |
